# Evaluation of Women Presenting With Bleeding in the First Trimester of Pregnancy in a Tertiary Hospital: A Cohort Study

**DOI:** 10.7759/cureus.95755

**Published:** 2025-10-30

**Authors:** Sangeetha V, Vidhya Selvam, Niveditha Prasath, Manoshi Chouhan

**Affiliations:** 1 Obstetrics and Gynaecology, Sree Balaji Medical College and Hospital, Chennai, IND

**Keywords:** adverse pregnancy, fetal outcomes, first-trimester bleeding, gestational hypertension, neonatal complications, preterm birth

## Abstract

Introduction

First-trimester bleeding ranges from harmless spotting to signs of serious complications such as miscarriage, ectopic pregnancy, and molar pregnancy. It is associated with adverse maternal outcomes, including gestational hypertension and preeclampsia, as well as neonatal risks like preterm birth and low birth weight. Understanding the risk factors and outcomes can improve the monitoring and management of affected pregnancies.

Methodology

This prospective cohort study included 110 pregnant women presenting with first-trimester vaginal bleeding at a tertiary hospital. Participants were followed through pregnancy to record maternal and neonatal outcomes. Risk factors such as diabetes, hypertension, and thyroid disorders were analyzed. Bleeding severity was categorized, and its association with pregnancy outcomes was evaluated using statistical analysis to identify significant correlations.

Results

Among the 110 participants, 24 (21.8%) experienced spontaneous abortion, 26 (23.6%) had preterm births, and 60 (54.5%) delivered at term. Gestational hypertension was observed in 16 women (14.5%, p=0.038), and preeclampsia in 12 women (10.9%, p=0.047), both significantly associated with first-trimester bleeding. Among the 86 live births, neonatal complications included low birth weight in 25 neonates (29.1%), neonatal intensive care unit (NICU) admissions in 20 neonates (23.3%), and APGAR scores <7 at five minutes in 12 neonates (14.0%). The severity of bleeding was directly related to adverse outcomes: among 26 women with heavy bleeding, preterm birth occurred in nine (34.6%), gestational hypertension in five (19.2%), NICU admission in 10 (38.5%), and low birth weight in 11 (42.3%) (all p<0.05). Conversely, women with mild spotting (n=46) generally had more favorable outcomes, with 33 (70.7%) achieving term delivery.

Conclusion

First-trimester bleeding is a significant clinical concern linked to increased maternal and neonatal complications, especially when bleeding is moderate to severe. Identifying associated risk factors and closely monitoring affected pregnancies allows for timely interventions, improving maternal and fetal outcomes, and reducing adverse effects.

## Introduction

Bleeding during the first trimester is a common occurrence, affecting approximately 15-25% of all clinically recognized pregnancies. The severity can range from mild spotting to significant vaginal bleeding. The extent and timing of the bleeding, in combination with other potential risks, are important factors in understanding how the pregnancy will progress. While it can be due to harmless causes, it may also indicate serious problems such as threatened miscarriage or ectopic pregnancy. Therefore, a quick assessment is necessary to ensure proper care, timely treatment if needed, and to provide reassurance for both patients and doctors [1].

The first trimester of pregnancy is very crucial for the baby's growth. Vaginal bleeding during this time can have various consequences, from a healthy pregnancy to a higher chance of miscarriage, preterm labour, or other problems. Researching these outcomes helps doctors provide personalized treatment and better monitor the pregnancy [2].

Although bleeding in the first-trimester of pregnancy is often harmless, it can sometimes indicate complications that need medical attention. Certain things can make complications more likely, such as being an older mother (over 35), having comorbidities like diabetes or high blood pressure, the use of fertility treatment, and unhealthy behaviors like smoking and drinking alcohol. Uterine problems, like fibroids, can also raise the risk. Knowing these factors helps doctors to carefully handle pregnancies with early bleeding, leading to better results through focused observation and treatment [3].

First-trimester bleeding is correlated with adverse maternal complications, such as placenta previa, placental abruption, and hypertensive disorders in pregnancy. Furthermore, it demonstrates an association with a high risk of preterm birth, low birth weight, and neonatal intensive care unit (NICU) admission. While not invariably predictive of negative events, the recognition of these associations allows for intensified surveillance and prompt therapeutic strategies to optimize maternal and neonatal well-being [4].

Although progress has been made in obstetric care, the etiology of first-trimester hemorrhage is largely unexplained beyond its established correlation with spontaneous abortion. Recent investigations suggest an association between early pregnancy bleeding and increased cesarean delivery rates, as well as subsequent maternal morbidity. This highlights the critical requirement for exhaustive studies evaluating the enduring consequences on maternal and offspring well-being. Enhanced comprehension of this phenomenon will permit healthcare providers to formulate refined clinical protocols, thereby promoting enhanced care and superior prognoses for mothers and their infants across the continuum of pregnancy and puerperium [5].

First-trimester bleeding frequently induces considerable distress and apprehension regarding potential pregnancy termination, which can negatively affect both maternal and fetal health. Empathetic guidance, clear communication, and holistic support are crucial in mitigating these anxieties. Providing women with knowledge pertaining to possible causes and prognoses facilitates informed choices and guarantees suitable surveillance and prompt treatment to optimize health outcomes [6].

Previous research has identified that first-trimester bleeding may be associated with adverse pregnancy outcomes such as spontaneous abortion, preterm delivery, and low birth weight; however, the extent to which maternal and obstetric factors predict these outcomes remains inadequately understood. The present study addresses this gap by evaluating the feto-maternal outcomes and identifying risk factors among women presenting with first-trimester bleeding in a tertiary hospital setting. The findings demonstrate that while many pregnancies with early bleeding progress uneventfully, the risk of miscarriage and adverse maternal outcomes increases significantly among women with heavy bleeding, low socioeconomic status, and associated comorbidities. These insights contribute to improving early risk assessment and guiding timely obstetric intervention to optimise pregnancy outcomes [7].

For healthcare providers, it means refining diagnostic tools and management strategies to minimize risks. For expectant mothers, it means receiving timely and accurate information that can ease fears and promote healthy pregnancy behaviors. Ensuring that women experiencing first-trimester bleeding receive appropriate evaluation and support throughout pregnancy is essential to improving maternal and fetal outcomes [8].

While many cases resolve without complications, others require careful monitoring and intervention. By closely examining the feto-maternal outcomes associated with early pregnancy bleeding, this study aims to provide essential insights that will guide clinical decision-making and enhance pregnancy care. With a deeper understanding of the causes, risk factors, and long-term implications of first-trimester bleeding, healthcare providers can offer more effective support, ensuring the best possible outcomes for both mothers and their babies [9]. This study aimed to evaluate women presenting with bleeding in the first trimester at a tertiary hospital, to analyze the associated feto-maternal outcomes, and to identify risk factors associated with the bleeding.

## Materials and methods

Study design

This was a prospective, observational cohort study. The design was chosen to enable real-time tracking of maternal and fetal outcomes in women who presented with first-trimester bleeding, while minimizing recall bias and ensuring that events were captured as they unfolded. The prospective cohort approach provided an opportunity to establish temporal relationships between exposure to first-trimester vaginal bleeding and subsequent adverse outcomes during pregnancy, delivery, and the postpartum period. By enrolling participants at the time of presentation and following them systematically until delivery and recovery, the study design ensured robust longitudinal data collection and reduced the risk of missing crucial clinical information.

Study setting and duration

The study was conducted in the Department of Obstetrics and Gynaecology at Sree Balaji Medical College and Hospital (SBMCH), Chennai, Tamil Nadu, India. Being a tertiary care teaching hospital, SBMCH caters to a heterogeneous patient population from both urban and semi-urban areas, ensuring a representative sample of women presenting with first-trimester bleeding. The institution offers comprehensive diagnostic and management facilities, including ultrasonography, biochemical testing, and emergency obstetric care, facilitating systematic evaluation and follow-up of all participants.

Participant enrolment was carried out over a period of 12 months, from July 2023 to June 2024, and data collection continued for an additional nine months to ensure complete follow-up through delivery and the early postpartum period for all enrolled participants. This extended timeline minimised loss to follow-up and allowed for comprehensive outcome assessment.

Study population

The study population included pregnant women presenting to the outpatient or emergency department with complaints of vaginal bleeding during the first trimester, defined as a gestational age up to 12 completed weeks. Only women with confirmed singleton intrauterine pregnancies were eligible. Participants were recruited consecutively at their first presentation following eligibility screening. All enrolled women were counseled and encouraged to deliver at SBMCH to ensure uniformity of data collection. However, those who delivered elsewhere were contacted telephonically and through outpatient follow-up to obtain delivery and neonatal outcome information. Participants lost to follow-up despite these efforts were excluded from outcome analysis but included in baseline descriptive statistics to maintain transparency in reporting.

Inclusion and exclusion criteria

To maintain scientific rigor, strict inclusion and exclusion criteria were applied. Women were eligible if they had confirmed singleton intrauterine pregnancies and presented with vaginal bleeding within the first trimester, beginning from conception and extending up to 12 weeks of gestation. Exclusion criteria were carefully framed to eliminate confounding factors that could independently influence bleeding or pregnancy outcomes. Women with congenital or acquired bleeding disorders, such as hemophilia or von Willebrand disease, were excluded to avoid bias from systemic conditions that inherently predispose to hemorrhage. Similarly, women currently receiving anticoagulant therapy, such as heparin or warfarin, were excluded as their risk of bleeding was pharmacologically induced. Women who had ingested abortifacient drugs prior to presentation were also excluded since outcomes in this group would not reflect the natural course of first-trimester bleeding. In addition, multiple pregnancies, such as twin or higher-order gestations, were excluded due to their unique maternal and fetal risks, which could act as confounders in the analysis.

Sample size calculation

The sample size was calculated using Dobson’s formula to ensure adequate statistical power. The formula applied was $n = \frac{Z^2 \, P(1 - P)}{d^2}$, where Z represented the z-statistic for 95% confidence interval (1.96), P corresponded to the expected prevalence of first-trimester bleeding in previous studies (7.04%) [10], and d was the allowable error fixed at 10%. Based on these values, the sample size required was calculated to be 100 participants. To account for a potential 10% non-response rate, the final sample size was inflated to 110 participants. This approach ensured that the study was adequately powered to detect statistically significant associations between first-trimester bleeding and adverse outcomes.

Sampling method

The study employed a non-probability convenience sampling technique. Eligible participants presenting to the outpatient department or emergency services during the study period were recruited consecutively until the desired sample size was achieved. While probability sampling could have improved external validity, convenience sampling was justified due to the time-bound nature of the study and the relative rarity of first-trimester bleeding in the general obstetric population. Consecutive recruitment minimized selection bias within the hospital setting.

Data collection

Data were collected prospectively using a structured, pre-validated questionnaire administered at the time of enrollment and during subsequent follow-up visits. This instrument captured a wide range of variables spanning socio-demographic characteristics, detailed obstetric history, clinical presentation, ultrasound findings, laboratory parameters, and final pregnancy outcomes. Data collection was carried out by trained obstetric residents to ensure consistency and accuracy.

A comprehensive history was obtained from each participant, including maternal age, gravidity, parity, gestational age at the onset of bleeding, and previous obstetric history, particularly miscarriages, infertility, or adverse pregnancy outcomes. Potential risk factors such as smoking, chronic medical comorbidities, and a history of assisted reproductive technology (ART) use were also recorded. Following history taking, all participants underwent a detailed clinical examination, including general physical examination, abdominal palpation to assess uterine size and tenderness, and speculum examination to identify the source and quantify the severity of vaginal bleeding.

Ultrasound evaluation, using either transabdominal or transvaginal approaches, was performed in all participants to confirm fetal viability, determine gestational age, and assess placental position. The scans were also used to detect complications such as subchorionic hematoma, ectopic pregnancy, or gestational trophoblastic disease. Laboratory investigations included baseline tests such as complete blood count, blood grouping, and Rh typing, as well as thyroid function tests. Coagulation profiles were ordered selectively in women with heavy or recurrent bleeding or in cases where bleeding disorders were clinically suspected.

Follow-up and outcome assessment

Participants were followed up systematically at key intervals throughout pregnancy and into the postpartum period. During the first trimester, outcomes such as continuation of pregnancy, miscarriage, or persistence of bleeding were recorded. In the second trimester, participants were monitored for the development of hypertensive disorders of pregnancy, fetal growth restriction, or abnormal placentation. Third-trimester follow-up focused on identifying preterm labor, preeclampsia, and complications such as placenta previa or placenta accreta. At delivery, the mode of birth, whether vaginal or cesarean section, was documented, along with immediate maternal outcomes. Postpartum follow-up focused on complications such as hemorrhage and maternal recovery. The severity of vaginal bleeding during the first trimester was categorized into spotting, light bleeding, and heavy bleeding based on criteria described by the American College of Obstetricians and Gynecologists (ACOG) and corroborated by similar definitions in obstetric research [11].

Maternal outcomes of interest included the development of hypertensive disorders such as gestational hypertension or preeclampsia, abnormal placentation including placenta previa or abruption, mode of delivery, and postpartum complications including postpartum hemorrhage. Fetal and neonatal outcomes included preterm birth, defined as delivery before 37 completed weeks of gestation, low birth weight defined as less than 2500 grams, admission to the neonatal intensive care unit, and a composite measure of perinatal morbidity and mortality.

Ethical considerations

The study protocol was reviewed and approved by the Institutional Human Ethics Committee of SBMCH (approval number: 002/SBMCH/IHEC/2023/2033) prior to commencement. Informed written consent was obtained from all participants after a thorough explanation of the study’s objectives, procedures, and potential risks. Confidentiality was strictly maintained by anonymizing all personal identifiers in the dataset. The study adhered to the ethical principles outlined in the Declaration of Helsinki (2013 revision) and ensured that the rights, safety, and dignity of all participants were safeguarded at every stage.

Statistical analysis

Data entry was carried out using Microsoft Excel (Microsoft Corporation, Redmond, Washington, United States) and subsequently exported to IBM SPSS Statistics for Windows, version 25 (IBM Corp., Armonk, New York, United States) for analysis. Descriptive statistics were used to summarize baseline characteristics, with means and standard deviations (SDs) applied for continuous variables and frequencies with percentages reported for categorical variables. Inferential statistical methods were applied to assess associations and determine predictors. The Chi-square test was used to evaluate the association between first-trimester bleeding and categorical outcomes such as miscarriage, preterm birth, or hypertensive disorders. Binary logistic regression analysis was conducted to identify independent predictors of adverse maternal and fetal outcomes while adjusting for potential confounders. A p-value of less than 0.05 was considered statistically significant throughout the analysis.

## Results

**Figure 1:**
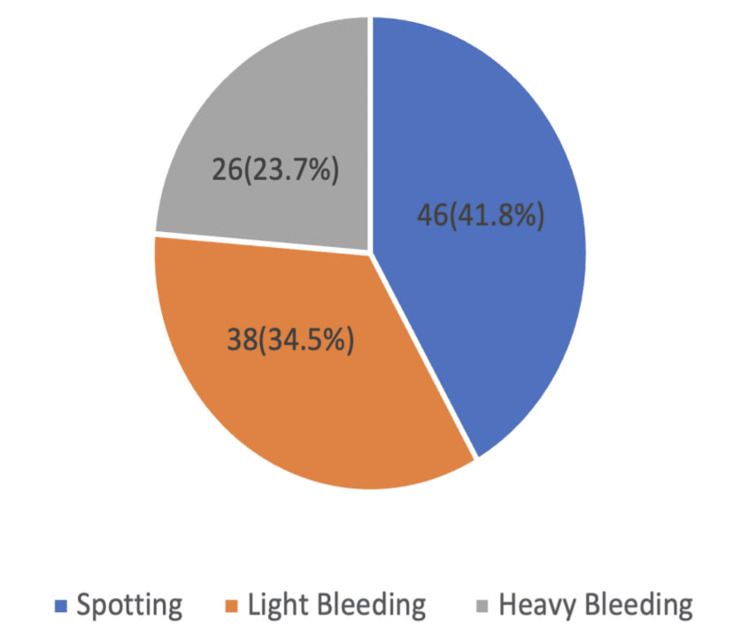
Distribution of first trimester vaginal bleeding among the study participants (N=110)

Among the 110 study participants, vaginal bleeding was categorized into three patterns based on the ACOG Practice Bulletin [11]. Spotting (defined as minimal blood loss, often seen only on wiping or as small spots on undergarments) was reported in 46 women (41.8%). Light bleeding (bleeding less than normal menstrual flow but requiring sanitary protection) occurred in 38 women (34.5%). Heavy bleeding (exceeding normal menstrual flow, often with passage of clots or requiring frequent pad changes) was noted in 26 women (23.6%). This is shown in Figure 1.

Among the 110 study participants, the majority were aged 21-30 years (n=68, 61.8%), followed by 31-40 years (n=30, 27.3%), while eight (7.3%) were <20 years and four (3.6%) were >40 years. Socioeconomic distribution showed that 37 (33.6%) belonged to the upper-lower class, 32 (29.1%) to the lower-middle, 21 (19.1%) to the upper-middle, and 20 (18.2%) to the lower class. Regarding parity, 70 (63.6%) were primigravida and 40 (36.4%) were multigravida. Risk factors included diabetes mellitus in 10 (4.6%), hypertension in five (2.3%), thyroid disorders in 12 (5.5%), and polycystic ovarian syndrome (PCOS) in 19 (8.7%). Based on BMI classification, 48 (43.6%) were obese, 32 (29.1%) overweight, 19 (17.3%) within the normal range, and 11 (10.0%) underweight (Table 1).

**Table 1:** Baseline characteristics of study participants (N = 110) PCOS: polycystic ovarian syndrome

Variable	Category	Frequency	Percentage
Age Group	<20 years	8	7.3
	21–30 years	68	61.8
	31–40 years	30	27.3
	>40 years	4	3.6
Socioeconomic Status	Upper Middle	21	19.1
	Lower Middle	32	29.1
	Upper Lower	37	33.6
	Lower	20	18.2
Parity	Primigravida	70	63.6
	Multigravida	40	36.4
Risk Factors	Diabetes Mellitus	10	4.6
	Hypertension	5	2.3
	Thyroid Disorders	12	5.5
	PCOS	19	8.7
BMI Category	Underweight (<18.5)	11	10.0
	Normal (18.5–24.9)	19	17.3
	Overweight (25–29.9)	32	29.1
	Obese (≥30)	48	43.6

Among the 110 participants, pregnancy outcomes included spontaneous abortion in 24 (21.8%), preterm birth in 26 (23.6%), and term pregnancy in 60 (54.5%). Maternal complications observed were gestational hypertension in 16 (14.5%), preeclampsia in 12 (10.9%), placenta previa in eight (7.3%), and placental abruption in four (3.6%) (Table 2).

**Table 2:** Pregnancy outcomes and maternal complications among study participants (N = 110)

Outcome / Complication	Frequency	Percentage
Pregnancy Outcomes		
Spontaneous Abortion	24	21.8
Preterm Birth	26	23.6
Term Pregnancy	60	54.5
Maternal Complications		
Gestational Hypertension	16	14.5
Preeclampsia	12	10.9
Placenta Previa	8	7.3
Placental Abruption	4	3.6

Among 86 live births, 25 (29.1%) neonates were of low birth weight, while 26 (30.2%) were delivered preterm. An APGAR score of less than 7 at five minutes was recorded in 12 (14.0%) newborns, and 20 (23.3%) required admission to the NICU (Table 3).

**Table 3:** Neonatal outcomes among babies born to mothers with first-trimester bleeding (N=86) NICU: neonatal intensive care unit

Neonatal Outcome	Frequency	Percentage
Low Birth Weight	25	29.10%
Preterm Birth	26	30.20%
APGAR <7 at 5 min	12	14.00%
NICU Admission	20	23.30%

Maternal and neonatal complications increased with the severity of vaginal bleeding. Among the 46 women with spotting, preterm birth occurred in seven (15.2%), hypertension in four (8.7%), NICU admission in five (10.9%), and low birth weight in eight (17.4%). In the 38 women with light bleeding, preterm birth was observed in nine (23.7%), hypertension in five (13.2%), NICU admission in eight (21.1%), and low birth weight in 10 (26.3%). Among the 26 women with heavy bleeding, preterm birth occurred in nine (34.6%), hypertension in five (19.2%), NICU admission in 10 (38.5%), and low birth weight in 11 (42.3%). These findings indicate a progressive increase in adverse maternal and neonatal outcomes with increasing severity of bleeding (Table 4).

**Table 4:** Distribution of maternal and neonatal complications by type of first trimester bleeding Chi-square test; *p-value < 0.05 is statistically significant NICU: neonatal intensive care unit

Complication	Spotting (n=46)	Light Bleeding (n=38)	Heavy Bleeding (n=26)	χ² value	p-value
Preterm Birth	7 (15.2%)	9 (23.7%)	9 (34.6%)	6.91	0.028*
Hypertension	4 (8.7%)	5 (13.2%)	5 (19.2%)	6.18	0.045*
NICU Admission	5 (10.9%)	8 (21.1%)	10 (38.5%)	9.36	0.012*
Low Birth Weight	8 (17.4%)	10 (26.3%)	11 (42.3%)	9.57	0.009*

## Discussion

First-trimester vaginal bleeding represents a significant clinical concern in obstetrics, often signaling potential complications for both mother and fetus. This prospective observational study, conducted at SBMCH, evaluated feto-maternal outcomes and associated risk factors in 110 women presenting with first-trimester bleeding. The study provides critical insights into demographic profiles, clinical risk factors, pregnancy outcomes, and complications, offering a robust basis for comparison with existing literature.

The age distribution of participants showed that 61.8% were aged 21-30 years, 27.3% were aged 31-40 years, with only a small proportion under 20 or over 40 years. This predominance of women in their prime reproductive years aligns with the findings of Hasan et al., who reported a high incidence of first-trimester bleeding in the 20-30 age group, attributing it to optimal fertility and higher pregnancy rates [3]. However, Saraswat et al. emphasized an elevated risk in women over 35 years due to age-related endometrial and vascular changes, a trend less pronounced in this study [10]. The limited representation of women over 40 may reflect regional demographic patterns or selection bias inherent to a tertiary care setting. Knudsen et al. further noted that both younger (<20 years) and older (>35 years) women are at increased risk for recurrent miscarriage, suggesting that age extremes warrant closer monitoring despite their lower prevalence in this cohort [12].

Parity analysis revealed that 63.6% of participants were primigravida, suggesting heightened vulnerability during first pregnancies. This finding is consistent with the findings of Yakıştıran et al., who linked primigravida status to increased bleeding risk due to suboptimal uterine receptivity or implantation challenges [1]. Conversely, Saravelos et al. highlighted that multiparous women with uterine anomalies or prior surgical scarring may also be predisposed to bleeding, a pattern less evident in this study [13]. The high proportion of primigravida women may reflect biological factors or the referral patterns of a tertiary care setting, emphasizing the need for further investigation into parity-specific risks.

Among clinical risk factors, diabetes mellitus (4.6%) and hypertension (2.3%) were significantly associated with first-trimester bleeding (p=0.01 for both), reinforcing the role of metabolic and vascular dysregulation in early pregnancy complications. Naskar et al. reported similar findings, noting that chronic conditions such as diabetes and hypertension impair placental vascularization, increasing the risk of bleeding [5]. Wendland et al. further highlighted the broader impact of gestational diabetes on pregnancy outcomes, suggesting that metabolic screening should be routine in women presenting with early bleeding [14].

Thyroid disorders and PCOS) did not reach statistical significance in this study, despite their established association with adverse outcomes. Du et al. observed that maternal hypothyroxinemia in the first trimester was not strongly linked to adverse outcomes, except for macrosomia, which may explain the lack of significance in this cohort focused on bleeding-related complications [15]. The non-significant findings for PCOS contrast with those of Stephenson and Kutteh, who identified PCOS as a risk factor for recurrent pregnancy loss, possibly due to hormonal imbalances affecting implantation [16]. The small sample size or effective clinical management of these conditions in the present study may account for these discrepancies, highlighting the need for larger studies to clarify their roles.

Lifestyle factors such as smoking, alcohol consumption, and high caffeine intake were not extensively analyzed due to low prevalence in this cohort. Nonetheless, prior studies by Castles et al. [17] and Greenwood et al. [18] established that smoking and excessive caffeine (>200 mg/day) impair placental development and increase miscarriage risk, underscoring the importance of systematic assessment of modifiable lifestyle factors. The limited reporting in this study may reflect cultural differences or underreporting, emphasizing the need for comprehensive history-taking in future research.

The study observed a spontaneous abortion rate of 21.8%, a preterm birth rate of 23.6%, and a term pregnancy rate of 54.5%. These outcomes differ slightly from prior reports, potentially reflecting improved antenatal care and population characteristics. Notably, the miscarriage rate is lower than meta-analytic reports, indicating nearly 50% miscarriage among women with first-trimester bleeding, suggesting a potential effect of timely intervention in this cohort. These outcomes align with van Oppenraaij et al., who reported increased risks of miscarriage and preterm labor in women with first-trimester bleeding, attributed to placental dysfunction [19]. Similarly, the miscarriage rate in this study is lower than that reported by Weiss et al., who found that nearly half of pregnancies with early bleeding result in loss, possibly due to differences in care practices or bleeding severity [20].

The high preterm birth rate (23.6%) corroborates findings by Goldenberg et al., who identified first-trimester bleeding as a significant risk factor for preterm labor due to inflammatory and vascular disruptions [21]. The spontaneous abortion rate also aligns with Everett’s prospective study, which reported a similar incidence of early pregnancy loss in women with bleeding before 20 weeks [22]. These consistent findings underscore the need for early intervention strategies, such as structured ultrasound monitoring and risk stratification, to optimize pregnancy outcomes.

Gestational hypertension and preeclampsia were observed in 14.5% and 10.9% of women, respectively, and were significantly associated with first-trimester bleeding. Placenta previa and placental abruption were less frequent but clinically relevant. These findings support the hypothesis that early bleeding reflects underlying placental dysfunction contributing to hypertensive disorders and abnormal placentation. Huang et al. further linked metabolic disturbances in early pregnancy to hypertensive disorders, suggesting that vascular pathology may underlie both early bleeding and subsequent complications [23].

Among 86 live births, 30.2% were preterm, 29.1% were low birth weight, 23.3% required NICU admission, and 14% had an APGAR score <7 at five minutes. These findings underscore the impact of first-trimester bleeding on fetal growth and perinatal morbidity, likely secondary to compromised placental function. The preterm birth and low birth weight rates are consistent with Stout et al., who linked early bleeding to neonatal morbidity through placental dysfunction and fetal hypoxia [24]. Badfar et al. further emphasized that maternal anemia, a potential confounder in bleeding cases, exacerbates fetal growth restriction, suggesting the importance of screening for anemia in this population [25]. The absence of stillbirths contrasts with the study of Flenady et al., who reported first-trimester bleeding as a risk factor for stillbirth, possibly due to timely interventions or the smaller sample size [26].

The findings underscore the importance of early ultrasound assessment, as advocated by Brown et al., to evaluate fetal viability and placental abnormalities [27]. Risk stratification of women presenting with first-trimester bleeding allows clinicians to implement close monitoring, individualized antenatal care, and timely interventions, potentially improving maternal and neonatal outcomes. Future research should focus on multicenter studies to validate these findings, explore long-term child health outcomes, and standardize bleeding classifications for uniform reporting.

This study has a few limitations to consider. Being conducted at a single tertiary care center, the findings may not be generalizable to broader populations or primary care settings. The sample size, while adequate for descriptive analysis, may limit the statistical power for detecting associations with less common risk factors. Lifestyle factors such as smoking, alcohol use, and caffeine intake were underreported, potentially introducing residual confounding. Additionally, first-trimester bleeding severity was self-reported, which may introduce reporting bias. Future studies incorporating multicenter cohorts, standardized bleeding quantification, and longitudinal follow-up would strengthen evidence and improve generalizability. The study did not assess the level or nature of physical activity among participants. However, physical exertion during the first trimester is typically low, and strenuous activities are generally discouraged during early pregnancy. This aspect should be explored in future studies to determine its potential influence on pregnancy outcomes.

## Conclusions

First-trimester vaginal bleeding serves as an important clinical marker associated with a higher risk of adverse maternal and neonatal outcomes, including preterm birth, hypertensive disorders of pregnancy, low birth weight, and increased rates of NICU admission. The presence of bleeding in early gestation reflects underlying placental or hormonal imbalances that can have long-term implications for pregnancy progression and fetal growth. Early identification and evaluation of both clinical and metabolic risk factors, such as maternal age, parity, body mass index, and glucose and hormonal profiles, are therefore critical for timely risk stratification.

Implementing structured monitoring protocols, such as serial ultrasound evaluations, biochemical screening, and individualized antenatal follow-up, can help in predicting and preventing complications. Integrating these measures within existing antenatal care frameworks will not only facilitate early intervention but also enhance maternal-fetal surveillance and improve perinatal outcomes. The study underscores the importance of adopting a multidisciplinary, evidence-based approach in the management of early pregnancy bleeding to reduce morbidity and mortality.
